# Cost-Effectiveness Analysis of Quadripolar Versus Bipolar Left Ventricular Leads for Cardiac Resynchronization Defibrillator Therapy in a Large, Multicenter UK Registry

**DOI:** 10.1016/j.jacep.2016.04.009

**Published:** 2017-02

**Authors:** Jonathan M. Behar, Hui Men Selina Chin, Steve Fearn, Julian O.M. Ormerod, James Gamble, Paul W.X. Foley, Julian Bostock, Simon Claridge, Tom Jackson, Manav Sohal, Antonios P. Antoniadis, Reza Razavi, Tim R. Betts, Neil Herring, Christopher Aldo Rinaldi

**Affiliations:** a Imaging Sciences & Biomedical Engineering, King’s College London, London, United Kingdom; b Department of Cardiology, Guy’s and St Thomas’ NHS Foundation Trust, London, United Kingdom; c St. Jude Medical, Stratford Upon Avon, United Kingdom; d Oxford Heart Centre, John Radcliffe Hospital, Oxford University Hospitals NHS Foundation Trust, Oxford, United Kingdom; e Great Western Hospital, Swindon, United Kingdom

**Keywords:** cardiac resynchronization therapy, cost-effectiveness, implantable cardiac defibrillator, left ventricular pacing, quadripolar lead, ACS, acute coronary syndrome, CRT, cardiac resynchronization therapy, CRTD, cardiac resynchronization defibrillator therapy device, HF, heart failure, ICER, incremental cost-effectiveness ratio, LV, left ventricular, NHS, National Health Service, NICE, National Institute for Health and Care Excellence, PNS, phrenic nerve stimulation, QALY, quality-adjusted life-year

## Abstract

**Objectives:**

The objective of this study was to evaluate the cost-effectiveness of quadripolar versus bipolar cardiac resynchronization defibrillator therapy systems.

**Background:**

Quadripolar left ventricular (LV) leads for cardiac resynchronization therapy reduce phrenic nerve stimulation (PNS) and are associated with reduced mortality compared with bipolar leads.

**Methods:**

A total of 606 patients received implants at 3 UK centers (319 Q, 287 B), between 2009 and 2014; mean follow-up was 879 days. Rehospitalization episodes were costed at National Health Service national tariff rates, and EQ-5D utility values were applied to heart failure admissions, acute coronary syndrome events, and mortality data, which were used to estimate quality-adjusted life-year differences over 5 years.

**Results:**

Groups were matched with regard to age and sex. Patients with quadripolar implants had a lower rate of hospitalization than those with bipolar implants (42.6% vs. 55.4%; p = 0.002). This was primarily driven by fewer hospital readmissions for heart failure (51 [16%] vs. 75 [26.1%], respectively, for quadripolar vs. bipolar implants; p = 0.003) and generator replacements (9 [2.8%] vs. 19 [6.6%], respectively; p = 0.03). Hospitalization for suspected acute coronary syndrome, arrhythmia, device explantation, and lead revisions were similar. This lower health-care utilization cost translated into a cumulative 5-year cost saving for patients with quadripolar systems where the acquisition cost was <£932 (US $1,398) compared with bipolar systems. Probabilistic sensitivity analysis results mirrored the deterministic calculations. For the average additional price of £1,200 (US $1,800) over a bipolar system, the incremental cost-effective ratio was £3,692 per quality-adjusted life-year gained (US $5,538), far below the usual willingness-to-pay threshold of £20,000 (US $30,000).

**Conclusions:**

In a UK health-care 5-year time horizon, the additional purchase price of quadripolar cardiac resynchronization defibrillator therapy systems is largely offset by lower subsequent event costs up to 5 years after implantation, which makes this technology highly cost-effective compared with bipolar systems.

## Graphical abstract

**Figure:**
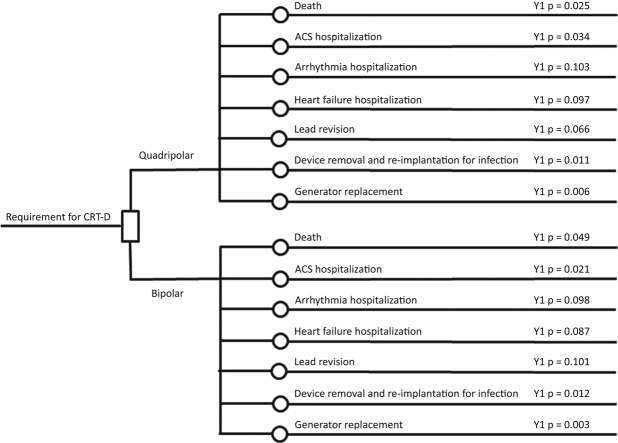

Cardiac resynchronization therapy (CRT) is an efficacious and cost-effective (1) treatment for patients with symptomatic heart failure with poor left ventricular (LV) function and prolonged QRS duration 2, 3, 4. Despite improvements in implantation delivery equipment and accumulation of user experience over the past 2 decades, approximately 30% of patients do not derive symptomatic benefit 5, 6. Post-implantation complications such as high capture thresholds, phrenic nerve stimulation (PNS), lead displacement, and infection reduce the effectiveness of this therapy 7, 8, 9, 10. The recent introduction of multipolar (quadripolar) LV leads has demonstrated a reduction in PNS through more proximal pole reprogramming, the presence of sustained lower capture thresholds, and easy deliverability (11).

However, new technology is usually provided at a higher purchase price than the conventional standard of care, which means that cost-effectiveness and affordability must be considered (12). Furthermore, the need for £22 billion in savings by 2020 in the United Kingdom (13) and an increased focus on efficiency as a result (14) further highlight the importance of cost-effective care. Multiple small clinical studies have demonstrated the clinical effectiveness of quadripolar leads at implantation and early follow-up 8, 15. Implant and 6-month follow-up data recently presented from the randomized MORE-CRT (More Options Available With a Quadripolar LV Lead Provide In-Clinic Solutions to CRT Challenges) trial (16) have confirmed the superiority of quadripolar leads, mainly from a reduction in intraoperative complications. We have previously demonstrated elimination of PNS and an associated lower all-cause mortality in patients implanted with a quadripolar lead in a large multicenter UK registry (17) (Online Figure 1).

We set out to assess the cost-effectiveness of quadripolar LV leads compared with bipolar LV leads in patients implanted with a cardiac resynchronization defibrillator therapy device (CRTD) within our previously published registry. We analyzed longer-term health-care utilization costs in terms of hospitalizations that occurred within the 5-year follow-up period to investigate whether the higher purchase price of this new technology was offset by expected reductions in cost arising from a reduction in hospitalizations. We also used mortality, acute coronary syndrome, and heart failure hospitalization data to estimate quality-adjusted life-year (QALY) differences.

## Methods

Clinical data were taken from a registry of patients with conventional CRT criteria who received device implants at 3 UK centers (Guy’s and St Thomas’ NHS Foundation Trust; John Radcliffe Hospital, Oxford University Hospital NHS Foundation Trust; and Great Western Hospital, Swindon) between January 2009 and January 2014. All patients provided fully informed consent. We have previously published the results of 5-year follow-up of patients, in which we compared patients with CRTD systems with a quadripolar versus a bipolar LV lead in terms of PNS, lead complications, and all-cause mortality (17).

For the purposes of the current study, hospitalization episodes for each patient in the clinical registry were reviewed and assigned to the following categories based on diagnosis: acute coronary syndrome (ACS), arrhythmia, heart failure hospitalizations, infection requiring system explantation and reimplantation, generator replacement, and revision of any lead. These were compared between patients implanted with a CRTD incorporating a quadripolar LV lead versus those with a bipolar LV lead. Quadripolar leads in the current analysis were exclusively the Quartet lead (St. Jude Medical, Sylmar, California). Only individuals with complete hospitalization data that included coding of the cause of hospitalization were included; as such, the cohort comprised 606 patients (quadripolar, n = 319; bipolar, n = 287).

**Figure 1:**
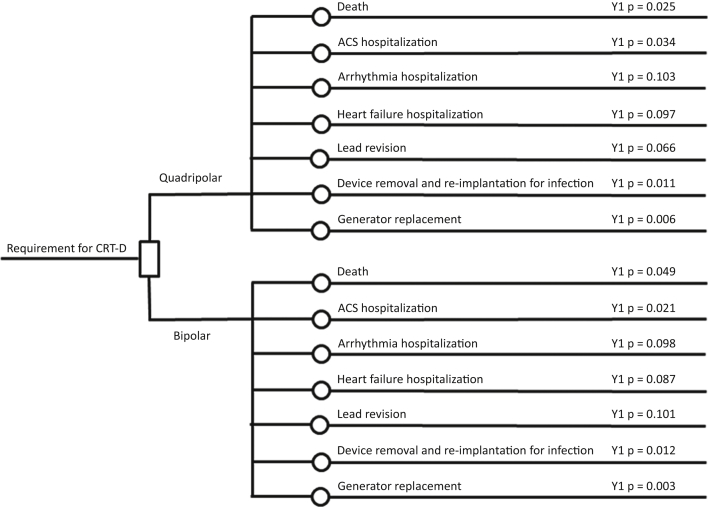
Model Diagram and Decision Structure Used in the Economic Model This was used for each of the 5 years, although only year 1 is shown here. Y1 p is the probability of the event in year 1; actual data for year 1 are shown. ACS = acute coronary syndrome; CRT-D = cardiac resynchronization defibrillator therapy device.

We performed an economic analysis of the registry data using all hospitalizations that occurred during the follow-up period. The rates of hospitalizations in each year from implantation were multiplied by the national tariff that pertained to the cause of hospitalization (Table 1). There was no extrapolation of data or event rates beyond the 5-year follow-up after implantation. Event rates were those that were observed to have occurred in each year; we did not derive transition probabilities that could be used for a Markov model. All events were counted, and some events occurred more than once in individual patients. A probabilistic sensitivity analysis was also undertaken to help understand the impact of parameter uncertainty and determine the probability that quadripolar CRTD was cost-effective. Probabilistic analysis was conducted by inputting data as probability (beta) distributions rather than point estimates and randomly sampling 1,000 values from these distributions. This was performed for all hospitalization episodes in addition to mortality data from our previous work (17). Comparative purchase costs were estimated between the quadripolar Quartet leads (St. Jude Medical) and the mean purchase cost of bipolar leads used in the clinical registry (QuickFlex, St. Jude Medical; AttainAbility, Medtronic; Easytrak, Boston Scientific). A National Health Service (NHS, the UK health system) perspective was used, which means that wider societal impact was not considered. Costs and effects beyond year 1 were discounted at 3.5%, following the methodology recommended by the UK’s National Institute of Health and Care Excellence (NICE) (20). A model diagram (Figure 1) demonstrates the differing probabilities of hospitalization event rates (per cause) for year 1 post-implantation in those with quadripolar and bipolar systems. The same approach was used for years 2 to 5 in the analysis, and rates for all years are shown in Online Table 1.

### Costs

National tariff “enhanced tariff option” prices for 2015 to 2016 (18) were applied to ACS hospitalization, arrhythmia hospitalization, heart failure hospitalization, and lead revision procedures. The base tariff price was multiplied by the local cost factor (market forces factor) for each NHS hospital that implants CRT devices, and the mean of these values was used in the model. Table 1 shows the mean unit cost data used in the calculations per hospitalization, including local cost factors. Online Table 2 shows the equivalent costs in US dollars using a simple conversion of £1 = $1.50. Where there were different tariff values for elective/nonelective procedures and different values for complication/comorbidity splits, averages weighted by the number of admissions for each were calculated. Costs for CRTD implantation, device removal and reimplantation for wound infection, and CRT generator replacement were taken from the data used to inform the economic evaluation that underpinned NICE’s 2014 Technology Appraisal Guidance (19). The additional purchase cost of quadripolar technology was estimated to be £1,200 ($1,800) for the base-case analysis (market estimate, St. Jude Medical) but varied between zero and £2,400 ($3,600) to assess sensitivity, because acquisition price may vary according to local procurement arrangements. Quadripolar device removal and reimplantation for infection was uplifted by the additional acquisition cost for the quadripolar device, on the assumption that the same type of device would be reimplanted. Quadripolar generator replacement was costed at bipolar cost plus 0.67 of additional quadripolar system costs. Quadripolar lead revision was costed at bipolar cost plus 0.33 of additional quadripolar system costs.

### Quality-adjusted life years

The use of QALYs allows clinical effectiveness to be expressed in a common unit, to which a cost can be applied to estimate the value of health-care interventions. The EQ-5D questionnaire is commonly used to determine the quality-of-life utility values that can be translated into QALYs (21). Hawkins et al. (22) discussed this approach in the context of cardiac interventions, and it is a standard part of NICE’s methodology (20). The incremental cost-effectiveness ratio (ICER) is obtained by dividing the additional cost of using the new device by the incremental QALYs gained and can be used to estimate a value for decision-making purposes. NICE’s methods guide (20) suggests an ICER of £20,000 ($30,000) to £30,000 ($45,000) is the range in which cost-effectiveness is acceptable in terms of effective use of NHS resources; therefore, this was the benchmark used to assess the results of the current study.

**Figure 2:**
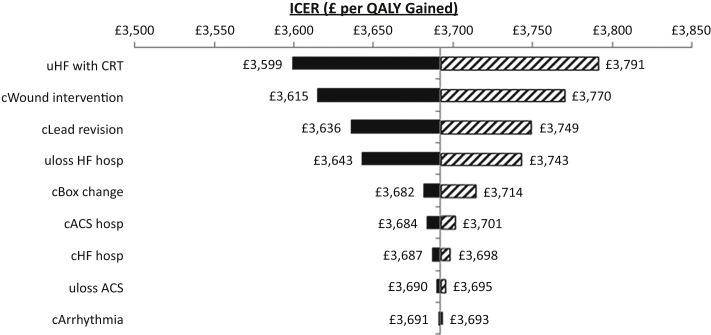
Tornado Plot Showing the Impact of Varying the Input Parameter Values to Their Upper and Lower 95% CIs on the Base-Case ICER **Hatched bars** show the impact of using the lower 95% confidence interval (CI); **solid bars** show the impact of using the upper 95% CI. Data labels on each **bar** show the incremental cost-effectiveness ratio (ICER) resulting from the change in value. A shift to the **right of the center line** shows an ICER that denotes less favorable cost-effectiveness than the base case. ACS = acute coronary syndrome; c = cost of; HF = heart failure; hosp = hospitalization; QALY = quality-adjusted life-year; quad = quadripolar cardiac resynchronization therapy system; u = utility value.

Only the mortality difference used in our previous report (17), utility loss attributable to ACS events, and utility loss attributable to heart failure hospitalizations were used to assess QALY differences between bipolar and quadripolar devices, similar to the methods used in the economic analysis that informed NICE’s recent technology appraisal of implantable cardioverter-defibrillators and CRT (19). A baseline EQ-5D utility of 0.8808 was used for a patient with heart failure and a CRT device (range: 0.85 to 0.903), with utility loss because of death being taken as a loss from this value to zero. The utility loss associated with a heart failure admission was calculated, from the work of Swinburn et al. (23) and Lewis et al. (24), to be 0.1197, persisting for 18 days (average length of stay plus 7 days post-discharge). The utility loss associated with ACS events was calculated, from the work of Lewis et al. (24) and Matza et al. (25), to be 0.1035, persisting for 10.4 days (average length of stay plus 7 days post-discharge). A range of input parameters were varied by ±95% confidence interval to show the impact of each on the base-case ICER, and the results are shown on a tornado plot (Figure 2).

### Statistical analysis

Continuous variables are expressed as mean ± SD. Comparisons were made with a Student *t* test. Categorical data were expressed as an absolute number of occurrences and associated frequency (%); analysis was performed with a chi-squared test. A probability value of <0.05 was considered significant. Statistical analysis was performed with the Statistics Package for the Social Sciences (SPSS) version 21 (SPSS Inc., Chicago, Illinois). Economic analysis was undertaken in Microsoft Excel.

## Results

A total of 606 patients were included in this analysis and were matched with regard to age and sex. Patients in the bipolar group had a higher prevalence of ischemic heart disease (quadripolar vs. bipolar: 181 [56.7%] vs. 190 [66.2%]; p = 0.02), and fewer were in sinus rhythm (quadripolar vs. bipolar: 303 [95.0%] vs. 48 [83.3%]; p < 0.001) before implantation. Mean QRS duration was similar between groups (159 ± 6.2 ms vs. 160 ± 5.1 ms, quadripolar vs. bipolar, respectively; p = 0.07), as was the proportion of patients in New York Heart Association functional class III (183 [76.9%] vs. 145 [72.1%], quadripolar vs. bipolar, respectively; p = 0.10). Mean percentage of biventricular pacing throughout the follow-up period was similar between groups (Q: 94.6 ± 1.6% vs. B: 94.4 ± 1.5%, p = 0.11). Length of stay after implantation was similar between groups, irrespective of whether they were elective admissions (1.2 ± 2.3 days vs. 1.2 ± 1.6 days, quadripolar vs. bipolar, respectively; p = 1.00) or existing inpatients (5.0 ± 8.5 days vs. 5.2 ± 7.2 days, quadripolar vs. bipolar, respectively; p = 0.76), as shown in Table 2.

Patients implanted with a quadripolar lead had a significantly lower absolute number of all-cause hospitalizations (quadripolar: 191 admissions among 309 patients; bipolar: 225 admissions among 287 patients; p < 0.001), as shown in Table 3. Moreover, the proportion of patients hospitalized at least once was also significantly lower in those implanted with a quadripolar compared with a bipolar lead (42.6% vs. 55.4%, respectively; p = 0.002), as shown in Table 4. This was primarily driven by a significantly lower number of hospitalizations for heart failure (51 admissions among 309 patients with a quadripolar device vs. 75 among 287 patients with a bipolar device; p = 0.003) and CRTD generator replacement (9 admissions among 309 patients vs. 19 among 287 patients, respectively; p = 0.03). Hospitalizations for suspected ACS, arrhythmia, device explantation, and lead revisions were similar between the groups (p = NS). Each hospitalization, irrespective of cause, was counted as a separate event (Table 3); these values were multiplied by the appropriate tariff (Table 1) to produce health-care utilization costs for each group over the 5-year period.

Table 4 represents the proportion of patients implanted with either quadripolar or bipolar leads who had at least 1 admission for the listed reasons. The absolute values for the hospitalization causes are therefore less than in Table 3, because each of the events are only counted once per patient. The proportions of patients hospitalized at least once for heart failure (8.8% [quadripolar] vs. 13.9% [bipolar]; p = 0.05) or generator replacement (2.5% [quadripolar] vs. 6.6% [bipolar]; p = 0.02) were significantly lower in those in whom a quadripolar lead was implanted. The average number of admissions for those who were hospitalized was similar in each group (1.40 vs. 1.42, quadripolar vs. bipolar).

### Cost-effectiveness analysis

**Figure 3:**
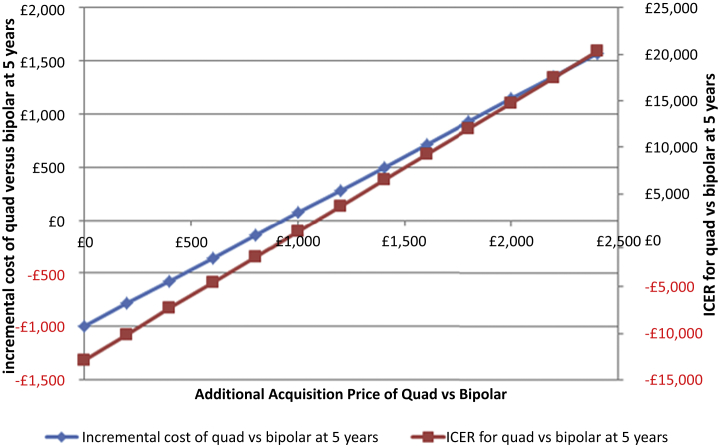
Incremental Cost and Cost-Effectiveness of Implanting a Quadripolar Versus Bipolar CRT-D System, Varied by the Additional Acquisition Cost of the Quadripolar System Quadripolar (quad) leads that cost up to £932 ($1,398) more than bipolar leads result in either a cost-neutral outcome or a cost saving because of reduced health-care utilization events. CRT-D = cardiac resynchronization defibrillator therapy device; ICER = incremental cost-effectiveness ratio.

The base-case ICER was £3,692 ($5,538) in the deterministic model (i.e., based on point estimates) and £3,835 ($5,753) in the probabilistic model. Up to an additional purchase cost of £932 ($1,398), quadripolar leads translated into a cumulative cost saving compared with bipolar leads because of the higher health-care utilization costs associated with the latter (Figure 3). The cost saving was up to £1,000 ($1,500) for purchasing a quadripolar system for the same price as a bipolar system (Table 5). Beyond £932 ($1,398), the additional ICER was up to £20,288 ($30,432) (Figure 3). Figure 2 shows the impact of varying a range of input parameters by ± 95% confidence intervals. The analysis was most sensitive to the utility of patients with heart failure, because death resulted in a loss of 0.8808 QALYs in each patient who died. All resulting ICERs remained <£4,000 per QALY gained.

**Figure 4:**
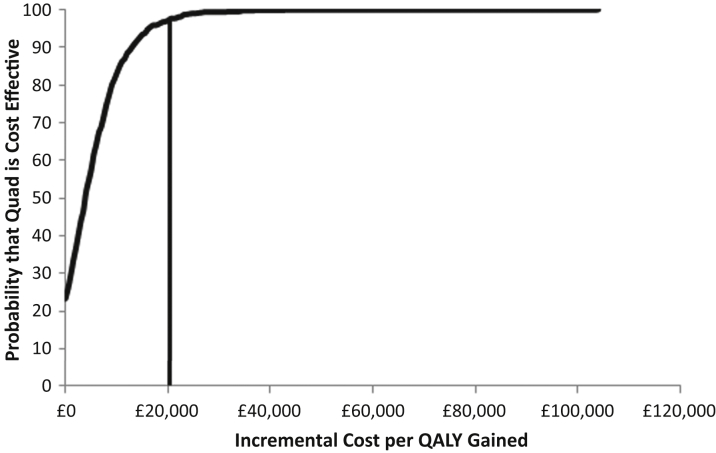
Cost-Effectiveness Acceptability Curve for Quadripolar Versus Bipolar CRTD The x-axis shows the willingness-to-pay threshold (i.e., the incremental cost per QALY gained). Quadripolar (Quad) CRTD is 97.1% likely to be cost-effective at £20,000 ($30,000) per QALY gained and 99.3% likely to be cost-effective at £30,000 ($45,000) per QALY gained. Abbreviations as in Figure 2.

**Figure 5:**
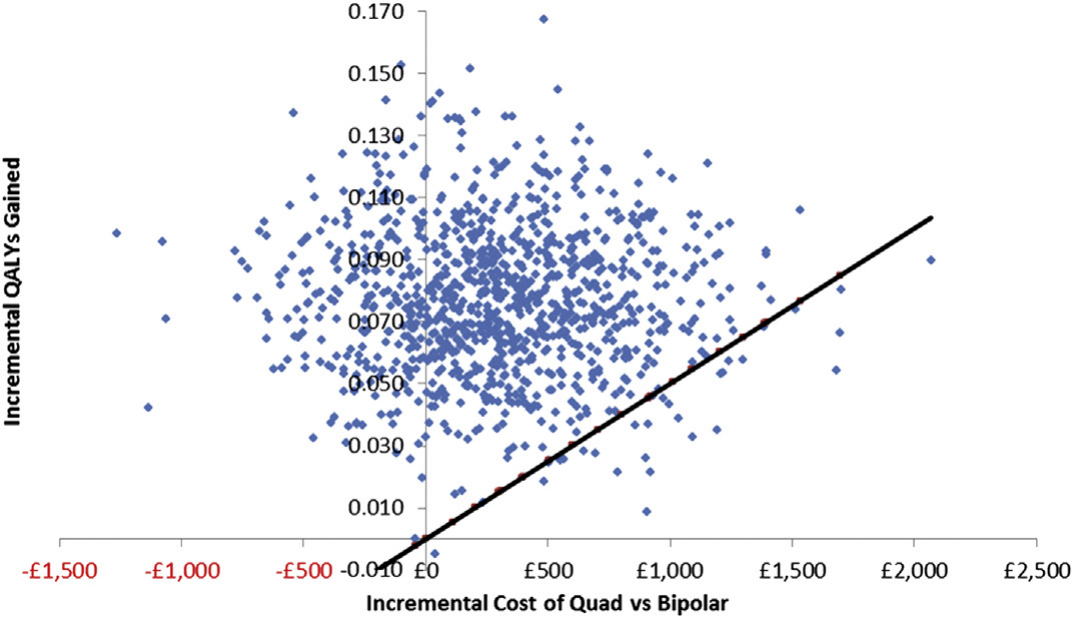
Cost-Effectiveness Plane Each point represents the result of 1 of the 1,000 simulations. Points to the left of the vertical axis are simulation results in which quadripolar CRTD was more effective and less expensive than bipolar CRTD. Points to the right of the vertical axis are simulation results in which quadripolar CRTD was more effective and more expensive than bipolar CRTD. The diagonal black line is the £20,000 ($30,000) per QALY gained line (i.e., all points above this are simulation results in which the incremental cost per QALY gained was <£20,000 [$30,000]). Abbreviations as in Figure 2.

In the probabilistic sensitivity analysis, quadripolar CRTD was 97.1% likely to be cost-effective at £20,000 per QALY gained and 99.3% likely to be cost-effective at £30,000 per QALY gained (Figure 4). A cost-effectiveness panel showing the results of each of the 1,000 simulations provides a visualization of the proportion of cases for which quadripolar systems were more effective and more expensive and for which they were more effective and less expensive (Figure 5).

## Discussion

This is the first comprehensive health economic analysis to use real-world UK clinical data from hospitalization events and mortality to produce an accurate comparison of cumulative cost differences between implanting quadripolar versus bipolar CRTD systems.

The main findings were as follows:

1. There was a lower absolute number of hospitalizations in patients in whom quadripolar CRTD systems were implanted, predominantly driven by a reduction in readmissions for heart failure and generator replacements.
2. Quadripolar CRTD systems, if purchased for up to £932 ($1,398) more than bipolar systems, yielded a cost saving over a 5-year period after health-care utilization costs were considered.
3. Quadripolar CRTD systems with an additional purchase price of £933 to £2,400 ($1,400 to $3,600) compared with bipolar systems remained cost-effective, with ICER values well within the range of acceptability used by NICE.
4. The calculated cost-effectiveness using real-world clinical data (deterministic model) was closely mirrored by the probabilistic sensitivity analysis, which reaffirms confidence in the results.

Multipolar LV leads for CRT delivery have demonstrated high implant success, good capture thresholds at implantation and follow-up, and a low rate of lead displacement 11, 26. Rates of intraprocedural lead complications appear lower than with conventional bipolar leads (27). Reduction or even elimination in PNS during medium-term follow-up provides invaluable utility in CRT delivery 9, 15. We have recently shown a reduction in all-cause mortality associated with quadripolar leads compared with a bipolar lead (17). Furthermore, rates of reintervention for lead repositioning were lower in those implanted with a quadripolar compared with a bipolar lead (2% vs. 5.2%; p = 0.03), and the radiation dose during implantation was almost one-half (1,028 cGy·cm^2^ vs. 1,950 cGy·cm^2^; p < 0.001).

The lower rates of hospitalization associated with a quadripolar lead in the current study could be driven by the improved efficacy in CRT delivery (attributable to PNS reduction and fewer reinterventions for lead displacement). Our previous study (17) also demonstrated lower implantation capture energy with quadripolar than with bipolar leads (0.95 μJ vs. 1.08 μJ; p = 0.003). Pacing systems consistently delivering higher-output voltages to capture the LV will have a reduced longevity (28), and this could explain the current findings of a significantly lower number of generator replacements among those implanted with a quadripolar system. This might be of significant clinical importance given the higher prevalence of device-related infections after generator replacement (29), which might contribute to greater morbidity and mortality in this group. Furthermore, from an economic modeling perspective, the lower proportion of generator changes in the quadripolar group was contributory to the lower overall health-care utilization costs over the follow-up period. The presence of 4 poles on the quadripolar LV lead allows greater programmability and the ability to choose multiple vectors for CRT delivery 30, 31. This provides the implanting physician with more choices for implantation locations, which might allow leads to be implanted more distally in posterolateral or lateral veins for stability purposes 8, 17, with the ability to stimulate the LV more basally from the proximal poles, which in turn could contribute to more optimal CRT delivery and could result in fewer heart failure hospitalizations and reduced mortality.

### Comparison with previous studies

Forleo et al. (32) have reported reduced rates of heart failure hospitalizations and LV lead revision among patients implanted with a quadripolar lead in a single-center Italian registry (events per patient per year: 0.15 vs. 0.32, quadripolar vs. bipolar; p = 0.04). Non–heart failure hospitalization rates were similar among groups. This study demonstrated lower health-care utilization costs associated with the quadripolar group (434 euros/patient-year vs. 1136 euros/patient-year; p = 0.02). However, the study by Forleo et al. (32) used Italian cost data that cannot be directly translated into the UK health-care setting, used only single-center data, had a much shorter follow-up period, included a smaller number of patients (193 vs. 606), and did not include as wide a range of clinical events in the follow-up costing. In the present study, the time to each event was calculated individually from the time of original implantation. In addition, the present study recorded and coded for all relevant acute and elective hospitalizations, not just heart failure and LV lead revisions, including admissions with ACS, arrhythmia, generator replacements, device extractions/reimplantation, and all right atrial and right ventricular lead revisions. Our calculated cumulative 5-year cost analysis was paralleled by the 5-year follow-up data, which provides confidence in the clinical relevance and accuracy of this study. Furthermore, we undertook a probabilistic sensitivity analysis similar to that by Forleo et al. (32), and the results for the base-case ICER closely mirrored the value calculated in the deterministic model. The only other contemporary UK-specific cost-effectiveness analysis was published recently by NICE (33). Recommendations made by NICE in a 2014 review of CRT and implantable defibrillators for heart failure were based on plausible ICERs in the range of £11,000 ($16,500) to £31,000 ($46,500) per QALY gained (19). By way of comparison, our results showed ICERs for quadripolar systems of up to £20,288 ($30,432) per QALY gained. Had data been available to include QALY adjustments for arrhythmia admissions, device removal and reimplantation for wound infection, generator changes, and lead revisions, it is likely that the ICERs would have been lower, because the rates of these events favor the quadripolar system. This analysis is therefore conservative with respect to the cost-effectiveness of quadripolar CRTD.

### Study limitations

The data used as the basis of this economic evaluation were derived from a multicenter clinical registry, and the choice of whether quadripolar or bipolar leads were implanted was not subject to a randomization process. However, the approach we have taken reflects current demands in which real-world data are becoming more important to assess the impact that new technologies have actually had on patients and health systems. We took real clinical events that occurred in NHS practice and applied NHS tariffs to them to determine the actual charge and cost-effectiveness. This was an in-study cost-effectiveness analysis, not an extrapolation to a lifetime horizon. We therefore did not assume event rates and did not model beyond the time for which we had gathered follow-up data. We did not perform a Markov model. Wider societal benefit was also not taken into account, which might be a further limitation.

As might be expected, the incremental acquisition cost of quadripolar technology is a strong determinant of the overall incremental cost-effectiveness of the 2 therapies. We therefore made an estimate of base cost and performed an analysis either side of the additional purchase cost to account for the variation in procurement acquisition costs. With respect to QALYs, the mortality difference was the strongest driver of the QALY gain associated with quadripolar CRTD. There was a significant difference in the proportions of patients with ischemic heart disease and those not in sinus rhythm (with more such patients in the bipolar group); however, this was corrected for in the multivariate analysis, and mortality remained significantly different.

## Conclusions

In a 5-year time horizon calculated from a UK health-care system perspective, the additional purchase price of quadripolar CRTD systems is substantially offset by lower health-care utilization costs, which suggests this technology is highly cost-effective compared with bipolar systems.

Perspectives **COMPETENCY IN MEDICAL KNOWLEDGE:** Quadripolar leads are associated with a lower rate of hospitalization than bipolar leads, primarily driven by fewer heart failure readmissions and generator replacements. This translates into a lower health-care utilization cost over a 5-year follow-up period and offsets the additional purchase price of quadripolar CRTD systems, which makes this technology highly cost-effective.

**TRANSLATIONAL OUTLOOK:** Given the near elimination of PNS, the reduction in hospital readmissions for generator replacement and heart failure, and the observed lower mortality in patients implanted with quadripolar leads, quadripolar CRTD systems may be considered the standard of care for CRT delivery.

## Figures and Tables

**Table 1:** National Tariff Tables: Hospitalization Pricing by Coding Category

Cost Item	Value (£)	Description	Source (Ref. #)
ACS hospitalization	3,421	EB10Z (actual or suspected MI), nonelective	ETO 2015–2016 (18)
Arrhythmia hospitalization	887	Activity-weighted average of EB07H (arrhythmia or conduction disorders with CC) and EB07I (arrhythmia or conduction disorders without CC)	ETO 2015–2016 (18)
Heart failure admission	2,756	Activity-weighted average of EB03H (heart failure or shock with CC) and EB03I (heart failure or shock without CC)	ETO 2015–2016 (18)
Lead revision procedure	2,952	Activity-weighted average of elective/nonelective HRG EA39Z (pacemaker procedure without generator implant; includes removal and reimplantation of cardiac pacemaker system)	ETO 2015–2016 (18)
Bipolar CRTD device	12,615		NICE technology appraisal (19)
Additional cost of quadripolar CRTD device	1,200	Base-case value, varied between £0 and £2,400 in sensitivity analysis	Market estimate 2015
Device removal and reimplantation for infection	23,506	Base value for bipolar device	NICE technology appraisal 2014 (19)
CRTD generator revision	15,990	Base value for bipolar device	NICE technology appraisal 2014 (19)

See Online Figure 1 for equivalent cost in US dollars.

ACS = acute coronary syndrome; CC = complications and comorbidities; CRTD = cardiac resynchronization defibrillator therapy device; ETO = extended tariff option (the national tariff scheme used by most English trusts in 2015–2016); MI = myocardial infarction; NICE = National Institute for Health and Care Excellence.

**Table 2:** Demographic Data

	Quadripolar (n = 319)	Bipolar (n = 287)	p Value
Age (yrs)	70.4 ± 11	68.7 ± 10	0.06
Female	50 (15.7)	48 (16.7)	0.74
Ischemic heart disease	181 (56.7)	190 (66.2)	0.02
Sinus rhythm	303 (95.0)	48 (83.3)	<0.001
QRS duration (ms)	159 ± 6.2 (n = 238)	160 ± 5.1 (n = 201)	0.07
NYHA functional class III symptoms	183 (76.9) (n = 238)	145 (72.1) (n = 201)	0.10
Mobitz II/complete heart block	9 (2.8)	14 (4.9)	0.21
% Biventricular pacing	94.6 ± 1.6	94.4 ± 1.5	0.11
LV lead upgrade	8 (2.5)	61 (21.3)	<0.001
Length of stay post-implantation (days) (elective)	1.2 ± 2.3	1.2 ± 1.6	1.00
Length of stay post-implantation (days) (inpatient)	5.0 ± 8.5	5.2 ± 7.2	0.76

Values are mean ± SD or n (%).

LV = left ventricular; NYHA = New York Heart Association.

**Table 3:** Absolute Numbers of Hospitalization, Split by Cause, and Corresponding Health-Care Costs∗

	Quadripolar (n = 319)		Bipolar (n = 287)		p Value
	n	Cost (£)	n	Cost (£)	
ACS	35	115,029	21	67,544	0.13
Arrhythmia	59	51,218	65	55,557	0.23
Heart failure	51	137,695	75	195,841	0.003
System explantation and reimplantation	5	121,122	6	136,788	0.76
Generator replacement	9	142,026	19	273,276	0.03
RA/RV lead revision	27	88,918	24	69,840	0.21
LV lead revision	5	16,466	15	43,650	0.02
Total episodes/cost	191	672,474	225	842,484	<0.001

Some patients were hospitalized for the same category more than once, and some not at all. The cost of events was calculated by multiplying the number of events in each year by the cost of the event for that year (i.e., events beyond year 1 were multiplied by the discounted cost for the year in which the event occurred).

ACS = acute coronary syndrome; LV = left ventricular; RA = right atrial; RV = right ventricular.

∗ Based on the tariff codes in Table 1.

**Table 4:** Numbers and Proportions of Patients in Each Group Who Have Been Hospitalized Once (or More)

	Quadripolar (n = 319)	Bipolar (n = 287)	Odds Ratio (95% CI)	p Value
ACS	26 (8.2%)	17 (5.9%)	1.40 (0.75–2.66)	0.34
Arrhythmia	39 (12.2%)	45 (15.7%)	0.75 (0.47–1.19)	0.24
Heart failure	28 (8.8%)	40 (13.9%)	0.59 (0.36–0.99)	0.05
System explantation and reimplantation	5 (1.6%)	6 (2.1%)	0.75 (0.23–2.47)	0.83
Generator replacement	8 (2.5%)	19 (6.6%)	0.36 (0.15–0.84)	0.02
Lead revision (RA/RV/LV)	30 (9.4%)	32 (11.2%)	0.83 (0.49–1.40)	0.50
Hospitalization (any cause)	136 (42.6%)	159 (55.4%)	0.59 (0.43–0.83)	0.002

Values are n (%).

CI = confidence interval; other abbreviations as in Table 3.

**Table 5:** Cumulative Total Cost of Implanting a Quadripolar Versus Bipolar CRTD for Different Acquisition Prices and Associated ICERs

Additional Acquisition Cost of Quadripolar CRTD (£)	5-Yr Incremental Cost of Quadripolar vs. Bipolar CRTD (£)	ICER of Quadripolar vs. Bipolar CRTD
0	−1,000	Quadripolar dominates
200	−786	Quadripolar dominates
400	−571	Quadripolar dominates
600	−357	Quadripolar dominates
800	−143	Quadripolar dominates
1,000	72	£926
1,200	286	£3,692
1,400	501	£6,458
1,600	715	£9,224
1,800	929	£11,990
2,000	1,144	£14,756
2,200	1,358	£17,522
2,400	1,572	£20,288

CRTD = cardiac resynchronization defibrillator therapy device; ICER = incremental cost-effectiveness ratio; Quadripolar dominates = quadripolar CRTD is less costly and more effective than bipolar CRTD at 5 years. In this situation, ICERs are negative and not conventionally shown.
