# Supplementary material for: Cost-Effectiveness Analysis of Quadripolar Versus Bipolar Left Ventricular Leads for Cardiac Resynchronization Defibrillator Therapy in a Large, Multicenter UK Registry
Source: JACC Clin Electrophysiol. 2017 Feb;3(2):107–16. doi: 10.1016/j.jacep.2016.04.009 (PMC5328196; doi:10.1016/j.jacep.2016.04.009)
Supplement: Online Figure 1 and Online Tables 1 and 2 [file mmc1.docx]

Online Figure 1

Reproduced with permission from Behar et al. (17)

Kaplan-Meier survival curves of patients implanted with a CRT. Quadripolar leads are denoted by the green line and bipolar leads by the blue line. Time from implantation on the x axis (days).


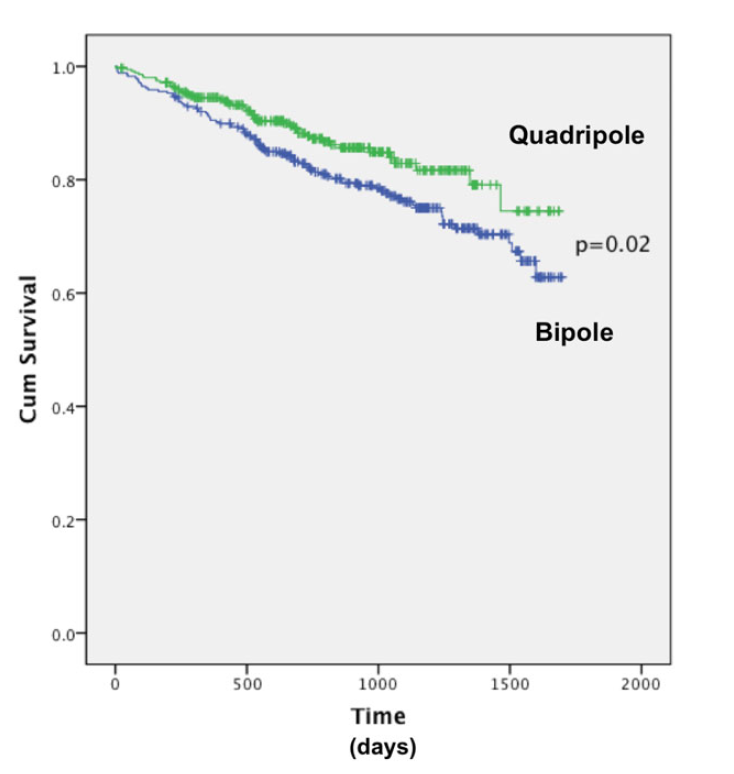


Online Table 1: Event rate data for all years.

|  |  | Year 1 | Year 2 | Year 3 | Year 4 | Year 5 |
| --- | --- | --- | --- | --- | --- | --- |
| Mortality | Quad | 0.025 | 0.025 | 0.031 | 0.025 | 0.025 |
|  | Bipole | 0.049 | 0.047 | 0.038 | 0.041 | 0.049 |
| ACS | Quad | 0.034 | 0.038 | 0.025 | 0.013 | 0.000 |
|  | Bipole | 0.021 | 0.007 | 0.017 | 0.024 | 0.003 |
| Arrhythmia | Quad | 0.103 | 0.053 | 0.022 | 0.006 | 0.000 |
|  | Bipole | 0.098 | 0.059 | 0.042 | 0.010 | 0.017 |
| HF admission | Quad | 0.097 | 0.041 | 0.013 | 0.009 | 0.000 |
|  | Bipole | 0.087 | 0.045 | 0.059 | 0.038 | 0.031 |
| Lead revision (any lead) | Quad | 0.066 | 0.022 | 0.013 | 0.000 | 0.000 |
|  | Bipole | 0.101 | 0.021 | 0.007 | 0.007 | 0.000 |
| Device removal and reimplantation for infection | Quad | 0.011 | 0.002 | 0.000 | 0.002 | 0.000 |
|  | Bipole | 0.012 | 0.005 | 0.002 | 0.000 | 0.002 |
| Generator replacement | Quad | 0.006 | 0.006 | 0.006 | 0.006 | 0.003 |
|  | Bipole | 0.003 | 0.007 | 0.007 | 0.017 | 0.031 |

Online Table 2. Equivalent tariffs in US dollars, using an average conversion factor of £1 = $1.50. We should note, however, that the health system within the US has very different absolute costs for the items described below. We have therefore only listed the US equivalent to the UK cost as per Table 1.

| Cost item | Value | Equivalent in US dollars |
| --- | --- | --- |
| ACS hospitalization | £3,421 | $5,132 |
| Arrhythmia hospitalization | £887 | $1,331 |
| Heart failure admission | £2,756 | $4,134 |
| Lead revision procedure | £2,952 | $4,428 |
| Bipolar CRTD device | £12,615 | $18,923 |
| Additional cost of quad CRTD device | £1,200 | $1,800 |
| Device removal and reimplantation for infection | £23,506 | $35,259 |
| CRTD generator revision | £15,990 | $23,985 |
